# Supplementary material for: Gut Microbiota Functional Dysbiosis Relates to Individual Diet in Subclinical Carotid Atherosclerosis
Source: Nutrients. 2021 Jan 21;13(2):304. doi: 10.3390/nu13020304 (PMC7911134; doi:10.3390/nu13020304)

# SUPPLEMENTAL MATERIAL

## GUT MICROBIOTA FUNCTIONAL DYSBIOSIS RELATES TO INDIVIDUAL DIET IN SUBCLINICAL CAROTID ATHEROSCLEROSIS


Andrea Baragetti[1*], Marco Severgnini[2*], Elena Olmastroni[3], Carola Conca Dioguardi[4], Elisa Mattavelli[1], Andrea Angius[5], Luca Rotta[6], Javier Cibella[7], Clarissa Consolandi[2], Liliana Grigore[8,9], Fabio Pellegatta[8,9], Flavio Giavarini[1], Donatella Caruso[1], Giuseppe Danilo Norata[1], Alberico L. Catapano[1,9§] and Clelia Peano[4,7§]

1. Department of Pharmacological and Biomolecular Sciences, University of Milan, Milan, Italy
2. Institute of Biomedical Technologies, National Research Council, Segrate, Milan, Italy
3. Epidemiology and Preventive Pharmacology Service (SEFAP), Department of Pharmacological and Biomolecular Sciences, University of Milan, Milan, Italy
4. Institute of Genetic and Biomedical Research, UoS Milan, National Research Council, Rozzano, Milan, Italy
5. Institute of Genetic and Biomedical Research, National Research Council, Cagliari, Italy
6. Department of Experimental Oncology, IEO, European Institute of Oncology, IRCCS, Milan, Italy
7. Genomic Unit, IRCCS, Humanitas Clinical and Research Center, Rozzano, Milan, Italy
8. S.I.S.A. Center for the Study of Atherosclerosis, Bassini Hospital, Cinisello Balsamo, Milan, Italy
9. Multimedica IRCCS, Milano Italy

*. These two authors equally contributed
§. These two authors are Senior authors

For correspondence:
Clelia Peano, PhD, Genomic Unit, Humanitas Clinical and Research Center, Via Manzoni 131, Rozzano, Milan, Italy. Tel +390282245146
Email: clelia.peano@humanitasresearch.it

Or

Alberico Luigi Catapano, Department of Pharmacological and Biomolecular Sciences – Università degli Studi di Milano
Via Balzaretti 9, 20133, Milan, Italy
e-mail: alberico.catapano@unimi.it


**Short title: GUT DYSBIOSIS AND SUBCLINICAL ATHEROSCLEROSIS**

EXPANDED METHODS........ 3
Serum zonulin and plasma TMAO quantifications........ 3
Full Statistical and Bioinformatic Data Analysis........ 3
*Microbiome 16S analysis........ 3*
*Metagenome analysis........ 4*
*Escherichia coli-associated L-carnitine degradation Pathway analysis........ 5*
EXPANDED RESULTS........ 6
Gut microbiota profiling and association with subclinical carotid atherosclerosis in the PLIC cohort........ 6
Analysis of the Gut Metagenome in subjects with advanced atherosclerosis progression........ 7
SUPPLEMENTAL REFERENCES........ 10
SUPPLEMENTAL TABLES........ 13
Supplemental Table 1 - Descriptives of the entire cohort divided by SCA stages........ 13
Supplemental Table 2 - Reduced and increased abundances of genera in SCA........ 15
Supplemental Table 3 - Daily intakes of food patterns reported by subjects of the studied population divided according to SCA........ 16
SUPPLEMENTAL FIGURES........ 18
Supplemental Figure 1 – GM Taxonomic relative abundances of phyla, families and genera in SCA. ........ 18
Supplemental Figure 2 - Accordance between 16s rRNA and shotgun metagenomic sequencing in the evaluation of relative abundances at the level of genera........ 19
Supplemental Figure 3 - Taxonomic markers associated with +IMT/+SCA........ 20
Supplemental Figure 4 – Correlation between most abundant bacteria and cardio-metabolic markers according to SCA........ 21
Supplemental Figure 5 – E. coli metagenetic markers associated with advanced SCA progression. ........ 22
Supplemental Figure 6 – Zonulin plasma levels are not increased in subjects with +IMT/+SCA........ 24

# EXPANDED METHODS

## Serum zonulin and plasma TMAO quantifications

Zonulin was measured from serum of subjects in the –IMT/-SCA and in those in the +IMT/+SCA group, using commercially available ELISA kit following the instructions of the manufacturer provided in the kit (cat#: EL-E-H5560, Elabscience, Whuan, Hubei, China).

TMAO was measured by stable isotope based liquid chromatography tandem mass spectrometry (LC/MS/MS) on plasma following sample processing and protocols as already described[S1].

## Full Statistical and Bioinformatic Data Analysis

### Microbiome 16S analysis

The 16S rRNA raw sequences were processed through paired-end reads merging by PANDAseq[S2] and discarding low quality reads (i.e., showing stretches of bases with a Q-score <3 for more than 25% of their length). In order to obtain a similar number of reads for each sample, a subset of 50,000 reads per sample was randomly extracted. Bioinformatic analyses were conducted using the QIIME pipeline (release 1.8.0[S3]); filtered reads were clustered into Operational Taxonomic Units (OTUs) at 97% similarity level and taxonomically assigned via the RDP classifier[S4] against the Greengenes database (release 13.8; (ftp://greengenes.microbio.me/greengenes_release/gg_13_8_otus), with a 0.5 identity threshold.

Biodiversity and distribution of the microorganisms were characterized via alpha- and beta-diversity evaluations. Alpha-diversity was measured using Chao1, observed species, Shannon diversity, Good's coverage and Faith's phylogenetic diversity (PD_whole_tree)

metrics; statistical evaluation among alpha-diversity indices was performed by a non-parametric Monte Carlo-based test, using 999 random permutations.

In order to compare the microbial community structure in beta-diversity analysis, we calculated weighted and unweighted UniFrac distances[S5] and performed principal coordinates analysis (PCoA); and statistical significance of the separation was assessed by analysis of variance with partitioning among sources of variation, using a permutation test with pseudo-F ratios (“adonis” function) in the R package “vegan” (version 2.0–10; https://cran.r-project.org/src/contrib/Archive/vegan/vegan_2.0-10.tar.gz).

Differences in abundances of bacterial taxa among experimental groups were analyzed by non-parametric Mann-Whitney U-test using MATLAB software (Natick, MA, USA). Unless otherwise stated, $p < 0.05$ were considered as significant for each statistical analysis.

**Metagenome analysis**

In order to remove low quality and human sequence, shotgun metagenomic reads were quality filtered using the human sequence removal pipeline (https://www.hmpdacc.org/hmp/doc/HumanSequenceRemoval_SOP.pdf) and the processing procedures (https://www.hmpdacc.org/hmp/doc/ReadProcessing_SOP.pdf) from the Human Microbiome Project[S6]. Resulting reads were, then, processed by HUMAnN2 pipeline (v. 0.11.2[S7]), which performs a nucleotide- and a translated-based search of reads against UniRef 90 protein database[S8]. Proteins were assigned to metabolic reactions and pathways via annotation on the MetaCyc pathways database of primary and secondary metabolism[S9]. In order to compensate for different sequencing depths, all measures were expressed as copies-per-million (CPM). Taxonomic profile of the samples were derived from the Metaphlan (v. 2.7.7[S10]) alignment within HUMAnN2 pipeline.

Alpha-diversity evaluation was performed on species-level taxonomic classification and MetaCyc reaction-level functional classification, using non-phylogenetic indexes (i.e.: Chao1, observed_species and Simpson's index) and a permutation-based Monte Carlo -based test with 999 random permutations in QIIME. Similarly, beta-diversity analysis was performed on Bray-Curtis and Euclidean distances and differences were assessed by "adonis" test with 999 random permutations in QIIME. Pathways were grouped to upper levels thanks to their lineage association in MetaCyc.

**Escherichia coli-associated L-carnitine degradation Pathway analysis**

Evaluation of *Escherichia coli*-associated L-carnitine degradation pathway was performed by retrieving CPM associated to the corresponding MetaCyc pathway ("CARNMET-PWY"). Since not all the genes in the pathway were consistently present, the "pathabundance" HUMAnN2 output was not usable. Thus, the corresponding records from the "genefamilies" output table of HUMAnN2, annotated to MetaCyc reactions and pathways were extracted. Briefly, from the default "genefamilies" CPM-normalized table, the "humann2_regroup_table" function was used to, first, regroup UniRef records to MetaCyc reactions ("RXN", using option "-g uniref90_rxn"), and, then, to the corresponding pathways (using option "-c $PYTHONPATH/humann2/data/pathways/metacyc_pathways"). Pathway annotation was added by "humann2_rename_table" function with "-n metacyc-pwy" option.

Abundance in genes belonging to the caiTABCDE operon of *E. coli* were retrieved searching for the corresponding UniRef_90 records (https://www.uniprot.org/uniref) and extracting the related CPMs from the "genefamilies" output table. Annotation of the records was manually checked, in order to consider only UniRef_90 records properly associated to *E. coli*. Significance of CPM differences was assessed by non-parametric Mann-Whitney U-test.

# EXPANDED RESULTS

## Gut microbiota profiling and association with subclinical carotid atherosclerosis in the PLIC cohort

The 16S amplicon sequencing, after quality filtering and subsampling, generated a total of 17,166,930 high-quality reads, with an average of 49,759 ± 2,142 reads per sample (range: 27,791 - 50,000 reads). This sequencing depth allowed the analysis and identification of the whole majority of the taxa diversity within the samples (Good's coverage index average: 0.94 ± 0.01).

The taxonomic profile of the cohort revealed a composition typical of Western individuals, confirming the relative abundances of the main phyla which compose Gut microbiota (e.g. Firmicutes (64.9%), Bacteroidetes (26.6%), Proteobacteria (3.4%), Verrucomicrobia (2.6%) and Actinobacteria (2.1%)).

Among families, Ruminococcaceae (32.9%) was the most abundant, followed by Lachnospiraceae (19.1%), Bacteroidaceae (15.6%), Veillonellaceae (4.3%) and Prevotellaceae (4.1%).

Most represented genera included Faecalibacterium (15.9% on average), Bacteroides (15.6%), Oscillospira (5.8%), Roseburia (4.7%), Prevotella (4.0%), Ruminococcus (3.8%), plus unidentified members of Ruminococcaceae and Lachnospiraceae families (rel. ab of 6.4% and 5.0%, respectively). The taxonomic profile of the cohort was in line with the composition already reported for western populations (**Supplemental Figure 1**).

Analysis of the genus-level relative abundances between the two groups highlighted a trend towards reduction of Bacteroides (average relative abundance: SCA: 14.6%; no SCA: 16.3%) and a significant increase of Escherichia (SCA: 2.8%; no SCA: 1.4%, p=0.008), Oscillospira (SCA: 6.5%; no SCA: 5.7%, p=0.013), as well as of Klebsiella

(**Supplemental Table 2**). The whole Enterobacteriaceae family resulted significantly increased in SCA individuals (p=0.002).

Notably, abundances of these same bacterial genera were not differently associated with anthropometric parameters (BMI, waist/hip ratio (WHR)), lipid profile, blood glucose (BGL), hematocrit and hs-CRP in subjects with SCA vs those without SCA. Any cluster of CVRFs correlated with alterations in GM composition in subjects with vs those without SCA (**Supplemental Figure 4**), we may suggest that dysbiosis in preclinical atherosclerosis is independent from the exposure to cardio-metabolic patterns.

## Analysis of the Gut Metagenome in subjects with advanced atherosclerosis progression

Shotgun sequencing produced an average of 66,571,289 ± 19,958,144 paired-end reads per sample (range: 12,548,694 - 106,635,518 reads), >99% of which were non-human; after duplicate removal, we obtained an average of 44,056,540 ± 12,622,793 usable reads (~66% of the initial reads) per sample, 54.5% of which were mapped on UniRef_90 database by HUMANn2 on average. Number of unmapped (i.e.: not matching Uniref_90 database) and unclassifiable (i.e.: having a UniProt identifier, but an unknown function) reads were comparable between classes (data not shown).

+IMT/+SCA metagenome composition significantly differed from that of the -IMT/-SCA in both alpha- (p=0.004 and p=0.001, for chao1 and observed_species metrics, respectively) and beta-diversity (p=0.002, p=0.004 and p=0.025 for bray-curtis, jaccard and euclidean distances, respectively).

Metagenome sequencing showed statistically significant differences in GM composition at all phylogenetic levels. In particular *Escherichia coli* (+6.7% on average) and several Streptococcus species (i.e.: *S. salivarius, S. parasanguinis, S. anginosus*) were increased

while members of Bacteroides genus, (i.e.: *B. uniformis* (-3.5% on average) and *B. thetaiotaomicron*) were reduced in +IMT/+SCA (**Table 2**).

Metagenome-derived taxonomies were highly consistent with those obtained with the 16S microbiota analysis of the same 46 subjects, showing a core set of differentially abundant genera identically found by both techniques, including the most abundant taxa (e.g.: Escherichia, Bacteroides, Blautia, Streptococcus); Dialister genus, on the other hand, was found as differentially abundant only by metagenome-derived taxonomic classification, with a notably discrepancy in -IMT/-SCA category (average difference of about 2%). Finally, genera found as differential in 16S sequencing only were all present at low abundances (<0.5% average relative abundance). Overall, Pearson's correlation confirmed that taxa abundances deriving from the two approaches were highly comparable ($\rho$=0.985) (**Supplemental Figure 2**).The observation that the biosynthesis of PE was the most up-represented in +IMT/+SCA subjects prompted us to focus on the L-carnitine degradation pathway, metabolically linked to L-carnitine/ γBB/trimethylamine (TMA) metabolic cascade 17,30. Abundance of reads mapping in genes belonging to the E. coli caiTABCDE operon was evaluated both in +IMT/+SCA and in –IMT/-SCA metagenomes (**Supplementary Figure 5, panel A**). The L-carnitine degradation pathway resulted significantly over-represented in +IMT/+SCA vs -IMT/-SCA (average CPM: 10.8±15.2 vs 1.1±2.3, p=0.007, Mann-Whitney U-test) and was almost entirely (~94%) due to E. coli contribution. The caiC, caiD and caiE genes were significantly upregulated in +IMT/+SCA individuals (log2 fold-changes of 3.3, 3.4 and 3.6 for caiC, caiD and caiE, respectively), whereas caiB showed a similar trend towards upregulation (log2 fold-change: 3.2). Notably, no associated counts were found on caiA or caiT genes.

**Validation of *E.coli* DNA abundance through a qPCR assay for the quantification of specific metagenomic markers**

To test whether *E. coli* caiD and caiE genes could be considered as markers of SCA progression, we designed and developed a specific qPCR assay to quantify the abundance of *E.coli* (**Supplementary Figure 5 panel A**) in the metagenomes of the -IMT/-SCA and the +IMT/+SCA phenotypes, and we observed a significant increase in their abundances in the latter (respectively P=0,028 and P= 0,014 for caiD and caiE) (**Supplementary Figure 5 panel B**). Moreover, among subjects with +IMT/+SCA, the increase in caiD and caiE abundances was associated with advanced SCA compared to subjects without SCA (p<0.001 for both caiD and caiE) (**Supplementary Figure 5, panel C-D**). Finally, the increase in caiD and caiE expression were not reflected by increased circulating plasma TMA N-Oxide (TMAO), a product of hepatic oxidation of TMA (16), in +IMT/+SCA compared to –IMT/-SCA subjects (**Supplemental Figure 5**).

**Circulating TMAO levels are not increased in presence of SCA**

Circulating plasma TMA N-Oxide (TMAO) was not increased in +IMT/+SCA compared to –IMT/-SCA subjects (11.64 (8.98-13.67) vs 12.78 (9.09-17.91) nM respectively, p=0.894) (**Supplementary Figure 5, panel E**), further supporting the presence of metagenetic markers of GM dysbiosis as earlier indicators of SCA progression.

## Supplemental references

**[S1]** Wang Z, Levison BS, Hazen JE, Donahue L, Li XM, Hazen SL. Measurement of trimethylamine-N-oxide by stable isotope dilution liquid chromatography tandem mass spectrometry. Anal Biochem. 2014;455:35–40.

**[S2]** Masella AP, Bartram AK, Truszkowski JM, Brown DG, Neufeld JD. PANDAseq: Paired-end assembler for illumina sequences. BMC Bioinformatics [Internet]. 2012 [cited 2020 Mar 30];13:31. Available from: http://www.ncbi.nlm.nih.gov/pubmed/22333067

**[S3]** Caporaso JG, Kuczynski J, Stombaugh J, Bittinger K, Bushman FD, Costello EK, Fierer N, Pẽa AG, Goodrich JK, Gordon JI, Huttley GA, Kelley ST, Knights D, Koenig JE, Ley RE, Lozupone CA, McDonald D, Muegge BD, Pirrung M, Reeder J, Sevinsky JR, Turnbaugh PJ, Walters WA, Widmann J, Yatsunenko T, Zaneveld J, Knight R. QIIME allows analysis of high-throughput community sequencing data. Nat. Methods. 2010;7:335–336.

**[S4]** Wang Q, Garrity GM, Tiedje JM, Cole JR. Naive Bayesian classifier for rapid assignment of rRNA sequences into the new bacterial taxonomy. Appl Environ Microbiol [Internet]. 2007 [cited 2019 Jul 6];73:5261–7. Available from: http://aem.asm.org/cgi/doi/10.1128/AEM.00062-07

**[S5]** Lozupone C, Lladser ME, Knights D, Stombaugh J, Knight R. UniFrac: an effective distance metric for microbial community comparison. ISME J [Internet]. 2011 [cited 2020 Mar 30];5:169–72. Available from: http://www.ncbi.nlm.nih.gov/pubmed/20827291

**[S6]** Methé BA, Nelson KE, Pop M, Creasy HH, Giglio MG, Huttenhower C, Gevers D, Petrosino JF, Abubucker S, Badger JH, Chinwalla AT, Earl AM, Fitzgerald MG, Fulton RS, Hallsworth-Pepin K, Lobos EA, Madupu R, Magrini V, Martin JC, Mitreva M, Muzny DM, Sodergren EJ, Versalovic J, Wollam AM, Worley KC, Wortman JR, Young SK, Zeng Q, Aagaard KM, Abolude OO, Allen-Vercoe E, Alm EJ, Alvarado L, Andersen GL, Anderson S, Appelbaum E, Arachchi HM, Armitage G, Arze CA, Ayvaz T, Baker CC, Begg L, Belachew T, Bhonagiri V, Bihan M, Blaser MJ, Bloom T, Bonazzi VR, Brooks P, Buck GA, Buhay CJ, Busam DA, Campbell JL, Canon SR, Cantarel BL, Chain PS, Chen IMA, Chen L, Chhibba S, Chu K, Ciulla DM, Clemente JC, Clifton SW, Conlan S, Crabtree J, Cutting MA, Davidovics NJ, Davis CC, Desantis TZ, Deal C, Delehaunty KD, Dewhirst FE, Deych E, Ding Y, Dooling DJ, Dugan SP, Michael Dunne W, Scott Durkin A, Edgar RC, Erlich RL, Farmer CN, Farrell RM, Faust K, Feldgarden M, Felix VM, Fisher S, Fodor AA, Forney L, Foster L, Di Francesco V, Friedman J, Friedrich DC, Fronick CC, Fulton LL, Gao H, Garcia N, Giannoukos G, Giblin C, et al. A framework for human microbiome research. Nature. 2012;486:215–221.

**[S7]** Franzosa EA, McIver LJ, Rahnavard G, Thompson LR, Schirmer M, Weingart G, Lipson KS, Knight R, Caporaso JG, Segata N, Huttenhower C. Species-level functional profiling of metagenomes and metatranscriptomes. Nat Methods [Internet]. 2018 [cited 2019 Jul 6];15:962–968. Available from: http://www.nature.com/articles/s41592-018-0176-y

**[S8]** Suzek BE, Wang Y, Huang H, McGarvey PB, Wu CH, UniProt Consortium. UniRef clusters: a comprehensive and scalable alternative for improving sequence similarity searches. Bioinformatics [Internet]. 2015 [cited 2020 Mar 30];31:926–32. Available from: http://www.ncbi.nlm.nih.gov/pubmed/25398609

**[S9]** Caspi R, Billington R, Fulcher CA, Keseler IM, Kothari A, Krummenacker M, Latendresse M, Midford PE, Ong Q, Ong WK, Paley S, Subhraveti P, Karp PD. The MetaCyc database of metabolic pathways and enzymes. Nucleic Acids Res [Internet]. 2018 [cited 2020 Mar 30];46:D633–D639. Available from: http://www.ncbi.nlm.nih.gov/pubmed/29059334

**[S10]** Segata N, Waldron L, Ballarini A, Narasimhan V, Jousson O, Huttenhower C. Metagenomic microbial community profiling using unique clade-specific marker genes. Nat Methods. 2012;9:811–814.

# SUPPLEMENTAL TABLES

## Supplemental Table 1 - Descriptives of the entire cohort divided by SCA stages.

Linear variables are reported as mean ± (standard error); dichotomous variables are reported as numbers (N) with percentage (%) out of total numbers within each SCA stage. "*" indicates P<0.010; "$" indicates P<0.050. Single "*" or "$" indicates significant difference between –IMT/-SCA vs +IMT/-SCA; Double "*" or "$" indicates significant difference between–IMT/-SCA vs -IMT/+SCA; Triple "*" or "$": comparison between –IMT/-SCA vs +IMT/+SCA. HDL-C: High Density Lipoprotein Cholesterol; LDL-C: Low Density Lipoprotein Cholesterol; ALT-alanine aminotransferase; AST-aspartate aminotransferase; GGT-Gamma-glutamyl transpeptidase; CPK-creatine kinase; IMT-Intima Media Thickness; "UI/L": International Units per Liter; Hs-CRP-High-sensitivity C-Reactive Protein; Mean Carotid IMT: the average value of ultrasound-derived IMT from right and left side of common carotid tract.

| | **"-IMT/-SCA" (N=23)** | **"+IMT/-SCA" (N=173)** | **"-IMT/ +SCA" (N=121)** | **"+IMT/+SCA" (N=23)** |
|---|---|---|---|---|
| Men, N(%) | 14 (60.87) | 51 (28.81) (*) | 45 (37.19) (**) | 14 (60.87) |
| Age (years) | 71.26 (7.02) | 62.84 (11.65) (*) | 71.22 (7.99) (**) | 76.39 (7.92) ($$$) |
| <=60 years-old | 4 (17.39) | 69 (38.98) | 11 (9.02) | 2 (8.7) |
| 60-70 years-old | 3 (13.04) | 61 (34.46) | 39 (31.97) | 2 (8.7) |
| 70-80 years-old | 15 (65.22) | 42 (23.73) | 60 (49.18) | 13 (56.52) |
| >80 years-old | 1 (4.35) | 5 (2.82) | 12 (9.84) | 6 (26.09) |
| Alcohol consumption, N(%) | 17 (73.91) | 112 (64.74) | 85 (69.67) | 17 (73.91) |
| Smoking habit, N(%) | 0 (0.00) | 22 (12.72) | 12 (9.84) | 3 (13.04) |
| Physical Activity, N(%) | 10 (45.45) | 105 (60.69) | 52 (42.62) | 8 (34.78) |
| BMI (Kg/m$^2$) | 26.36 (3.91) | 26.21 (3.85) | 26.69 (4.04) | 27.05 (3.69) |
| Lean, N(%) | 9 (40.91) | 67 (39.41) | 35 (29.66) | 3 (13.64) |
| Overweight, N(%) | 11 (50.00) | 75 (44.12) | 62 (52.54) | 17 (77.27) |

| | | | | |
|---|---|---|---|---|
| Obese, N(%) | 2 (0.09) | 28 (16.47) | 21 (17.80) | 2 (9.09) |
| Waist-hip Ratio | 0.91 (0.10) | 0.87 (0.08) (*) | 0.90 (0.08) | 0.93 (0.07) |
| Systolic pressure (mmHg) | 123.50 (8.45) | 125.38 (14.29) | 128.88 (15.46) | 131.7 (12.40) ($ $$) |
| Diastolic pressure (mmHg) | 75.43 (6.56) | 75.01 (9.00) | 76.50 (9.63) | 77.83 (8.44) |
| Antihypertensive, N(%) | 13 (56.52) | 60 (34.68) ($) | 68 (55.74) | 14 (60.87) |
| Total cholesterol (mg/dL) | 191.80 (21.95) | 204.34 (33.01) ($) | 193.12 (30.62) | 196.35 (43.52) |
| HDL-C (mg/dL) | 60.91 (12.03) | 62.40 (13.80) | 59.41 (13.55) ($$) | 52.39 (10.48) (***) |
| LDL-C (mg/dL) | 112.90 (18.07) | 122.27 (28.06) ($) | 113.83 (25.76) | 120.4 (37.11) |
| Triglycerides (mg/dL) | 90.22 (36.60) | 98.36 (36.78) | 99.32 (36.67) ($$) | 117.8 (47.63) ($ $$) |
| ApoA-I (mg/dL) | 160.00 (21.92) | 160.35 (20.07) | 155.44 (19.36) | 150.20 (16.05) |
| ApoB (mg/dL) | 96.65 (17.10) | 104.56 (26.13) | 97.16 (21.41) | 106.50 (32.12) |
| Lipid lowering drugs, N(%) | 13 (56.52) | 66 (38.15) | 71 (58.20) | 14 (60.87) |
| Fasting glucose (mg/dL) | 101.80 (10.79) | 100.11 (9.54) | 100.08 (12.20) | 102.30 (7.26) |
| Uric acid (mg/dL) | 5.31 (1.24) | 4.91 (1.36) | 5.42 (1.55) | 5.65 (1.33) |
| Serum creatinine (mg/dL) | 0.88 (0.21) | 0.81 (0.18) | 0.85 (0.20) | 0.91 (0.24) |
| ALT (UI/L) | 20.91 (11.24) | 21.73 (17.28) | 20.21 (11.14) | 21.57 (11.08) |
| AST (UI/L) | 22.87 (4.83) | 23.79 (6.57) | 23.47 (5.56) | 24.14 (5.38) |
| GGT (UI/L) | 22.04 (14.78) | 28.35 (39.86) | 25.02 (14.87) | 42.83 (79.07) |
| CPK (mg/dL) | 121.00 (56.23) | 121.03 (67.41) | 118.36 (58.21) | 131.60 (55.33) |
| Hs-CRP (mg/dL) | 0.07 (0.04-0.16) | 0.10 (0.50-0.20) | 0.11 (0.06-0.21) | 0.11 (0.06-0.32) |
| Neutrophils (cells*$10^3$/uL) | 3.33 (1.06) | 3.43 (1.24) | 3.48 (1.11) | 4.29 (1.85) ($$ $) |
| Lymphocytes (cells*$10^3$/uL) | 1.96 (0.54) | 2.01 (0.52) | 2.10 (1.34) | 2.16 (0.94) |
| Monocytes (cells*$10^3$/uL) | 0.54 (0.13) | 0.48 (0.14) | 0.53 (0.15) | 0.54 (0.15) |
| Eosinophils (cells*$10^3$/uL) | 0.16 (0.10) | 0.16 (0.10) | 0.17 (0.11) | 0.19 (0.10) |
| Basophils (cells*$10^3$/uL) | 0.04 (0.02) | 0.04 (0.019) | 0.04 (0.02) | 0.04 (0.02) |
| Mean Carotid IMT (mm) | 0.74 (0.11) | 0.71 (0.13) | 0.79 (0.12) (**) | 1.17 (0.21) (***) |

**Supplemental Table 2 - Reduced and increased abundances of genera in SCA.**

Relative abundances of the main bacterial genera in individuals with and without SCA. P-value refers to that of the two-sided Mann-Whitney U-test. For clarity, bacteria are grouped according to increase/decrease/unchanged status. *: P<0.05; **: P<0.01; ***: P<0.001.

| | Genus | avg. no SCA | avg. SCA | p-value |
|---|---|---|---|---|
| ▲ in SCA | Oscillospira | 5.356 | 6.486 | * |
| | Escherichia | 1.369 | 2.788 | ** |
| | Unclassified Christensenellaceae | 1.032 | 1.088 | * |
| | Klebsiella | 0.224 | 0.300 | *** |
| ▼ in SCA | Bacteroides | 16.311 | 14.579 | -- |
| | Unclassified Lachnospiraceae | 5.290 | 4.711 | -- |
| | Enterobacteriaceae (other) | 0.078 | 0.046 | *** |
| | Enterobacter | 0.023 | 0.018 | ** |
| | Schlegelella | 0.004 | 0.002 | ** |
| Unchanged | Faecalibacterium | 16.418 | 15.148 | -- |
| | Unclassified Ruminococcaceae | 6.125 | 6.837 | -- |
| | Roseburia | 4.999 | 4.262 | -- |
| | Unclassified Clostridiales | 4.795 | 5.387 | -- |
| | Ruminococcus | 3.591 | 3.932 | -- |
| | Prevotella | 3.452 | 4.725 | -- |
| | Lachnospira | 3.072 | 2.784 | -- |
| | Coprococcus | 2.908 | 2.357 | -- |
| | Akkermansia | 2.621 | 2.454 | -- |
| | Dialister | 2.388 | 1.859 | -- |
| | Unclassified Rikenellaceae | 2.241 | 2.601 | -- |
| | Parabacteroides | 1.544 | 1.588 | -- |
| | Bifidobacterium | 1.448 | 1.066 | -- |
| | Blautia | 1.218 | 1.256 | -- |

**Supplemental Table 3 - Daily intakes of food patterns reported by subjects of the studied population divided according to SCA.**

Intakes of food patterns (as g/week, based on the analysis of daily food diaries; see **Supplemental Methods**). "***" indicates P<0.005; "**" indicates P<0.01; "*" indicates P<0.05. P refers to that of the two-sided Mann-Whitney U-test. SD-standard deviation.

| | **no SCA** | **SCA** | |
|---|---|---|---|
| Food patterns (g/week) | **mean (DS)** | **mean (DS)** | **p** |
| Alcoholic beverages | 553.48 (797.43) | 548.65 (972.55) | 0.960 |
| **Beer, cider and substitutes** | 115.86 (279.71) | 94.39 (344.96) | 0.528 |
| Regular wine and substitutes | 431.04 (680.24) | 449.69 (777.31) | 0.815 |
| Spirits and liquors | 6.57 (21.54) | 4.57 (17.54) | 0.363 |
| Meat, meat products and substitutes | 385.27 (282.58) | 361.37 (322.81) | 0.470 |
| Beef, veal, poultry and pork, not preserved | 259.28 (213.11) | 249.19 (239.38) | 0.683 |
| Ham, salami, sausages and other preserved meats | 114.32 (113.74) | 104.17 (111.30) | 0.414 |
| Mechanically separated meats | 0.19 (1.52) | 3.60 (19.02) | 0.013 * |
| Offal, blood and their products | 5.22 (35.67) | 2.03 (14.76) | 0.316 |
| Cereal, cereal products and substitutes | 977.70 (640.34) | 791.39 (640.80) | 0.009 ** |
| Wheat, other cereals and flours | 64.18 (93.65) | 56.76 (92.11) | 0.469 |
| Bread | 284.94 (275.13) | 227.64 (257.15) | 0.053 |
| Pasta and pasta substitutes | 262.80 (212.73) | 239.90 (214.62) | 0.331 |
| Savory fine bakery products | 236.04 (253.42) | 147.94 (212.88) | 0.001 *** |
| Breakfast cereals | 124.37 (130.45) | 116.90 (134.28) | 0.608 |
| Processed cereals | 5.37 (23.77) | 2.25 (17.57) | 0.186 |
| Oils and fats | 90.89 (73.63) | 84.54 (77.27) | 0.444 |
| Butter and creams | 9.14 (20.09) | 8.11 (18.17) | 0.628 |
| Vegetable oils | 81.75 (67.12) | 76.43 (70.10) | 0.481 |
| Milk, milk products and substitutes | 855.92 (752.95) | 624.76 (681.00) | 0.004 *** |

| | | | |
|---|---|---|---|
| Milk and milk-based beverages | 560.95 (652.70) | 395.04 (575.58) | 0.016 * |
| Yoghurt and fermented milk | 132.21 (245.19) | 83.65 (181.88) | 0.047 * |
| Cheese and substitutes | 155.23 (147.76) | 136.66 (134.36) | 0.237 |
| Fruit, fresh and processed | 1071.33 (854.99) | 1184.27 (1054.22) | 0.278 |
| Fruits | 919.89 (777.56) | 1008.39 (913.73) | 0.338 |
| Processed fruits | 34.38 (99.69) | 37.47 (117.06) | 0.794 |
| Nuts | 13.86 (27.87) | 13.31 (27.03) | 0.857 |
| Dried Fruits | 2.51 (10.71) | 9.51 (40.29) | 0.020 * |
| Starchy Fruits | 100.70 (168.39) | 115.59 (228.73) | 0.490 |
| Pulses, fresh and processed | 61.08 (96.58) | 56.61 (112.99) | 0.696 |
| Fresh pulses | 6.96 (26.00) | 10.42 (48.76) | 0.399 |
| Processed pulses | 53.56 (95.72) | 44.68 (97.08) | 0.402 |
| Fish, seafood and fish products | 208.55 (227.48) | 164.72 (176.49) | 0.056 |
| Fish and seafood, fresh and frozen | 171.86 (216.57) | 133.76 (157.17) | 0.075 |
| Fish and seafood preserved | 36.68 (61.93) | 30.95 (57.75) | 0.388 |
| Fruit and vegetable juices | 101.66 (259.25) | 82.18 (254.52) | 0.492 |
| Eggs | 37.92 (58.62) | 41.04 (69.76) | 0.656 |
| Vegetables, fresh and processed | 930.78 (638.03) | 810.25 (720.47) | 0.105 |
| Leafy vegetables, fresh | 297.98 (285.09) | 242.24 (258.42) | 0.066 |
| Tomatoes, fresh | 83.82 (124.00) | 68.86 (116.74) | 0.262 |
| Other fruiting vegetables, fresh | 151.76 (199.43) | 129.34 (206.64) | 0.315 |
| Other vegetables | 215.43 (237.79) | 225.12(288.08) | 0.735 |
| Potatoes, tubers and their products | 120.33 (166.59) | 83.65 (122.82) | 0.027 * |
| Sweet products and substitutes | 127.37 (96.58) | 112.12 (153.60) | 0.327 |
| Sugar, fructose, honey and other nutritious sweeteners | 33.55 (56.28) | 26.24 (41.44) | 0.190 |
| Sweet products and substitutes | 93.83 (114.64) | 85.88(138.08) | 0.564 |

# SUPPLEMENTAL FIGURES

## Supplemental Figure 1 – GM Taxonomic relative abundances of phyla, families and genera in SCA.

Barplots of bacterial relative abundances at (A) phylum, (B) family and (C) genus level for the 345 PLIC individuals as estimated from 16S gut microbiota analysis. Each bar represents a sample. For clarity only the first 6, 21 and 24 most abundant phyla, families and genera, respectively, were represented. All remaining taxa were grouped into the "Other" category. Pie-charts on the right depict the average relative abundance for each taxa across all the samples.

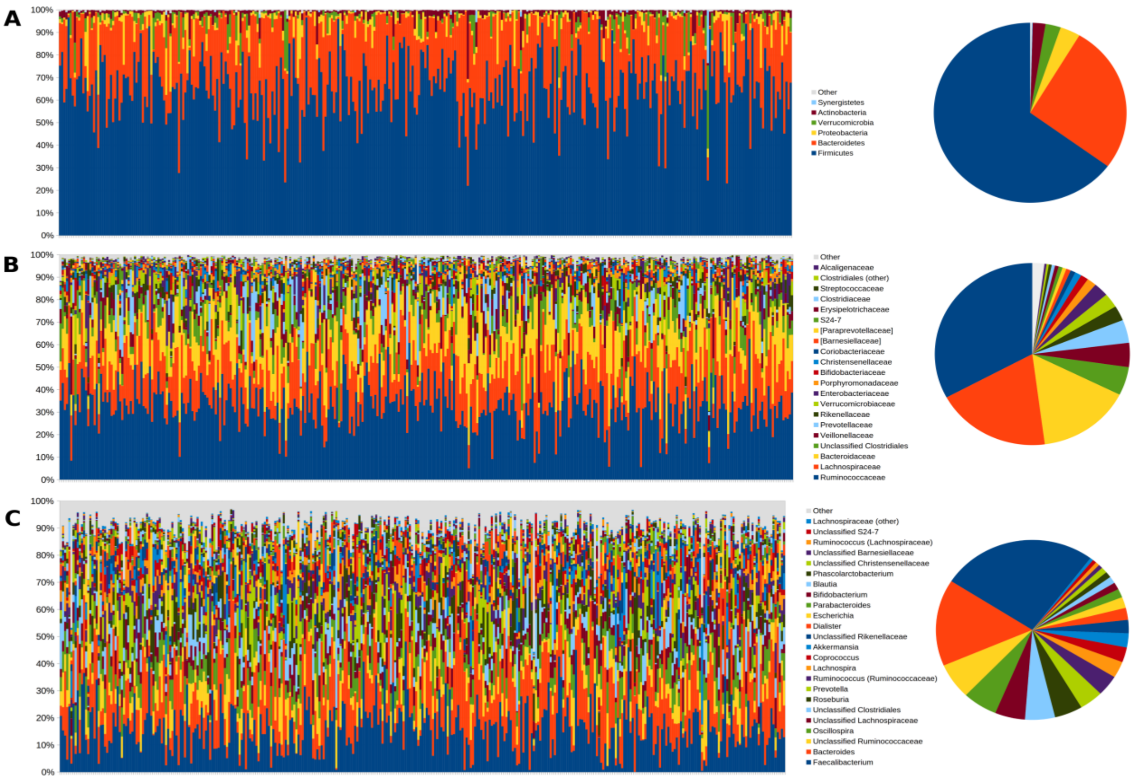

**Supplemental Figure 2 - Accordance between 16s rRNA and shotgun metagenomic sequencing in the evaluation of relative abundances at the level of genera.**

Line plots showing average relative abundance of taxa found as differential, divided for (A) “+IMT/+SCA” and (B) “-IMT/-SCA” individuals. Relative abundances are averaged for each genera and category (n=23). For each plot, relative abundances are depicted for metagenomics-derived (blue lines) and 16S-derived (red lines) sequencing. Dotted green line represents difference. Genera were grouped according to being found as differential by metagenome-only, 16S-only or both sequencing techniques.

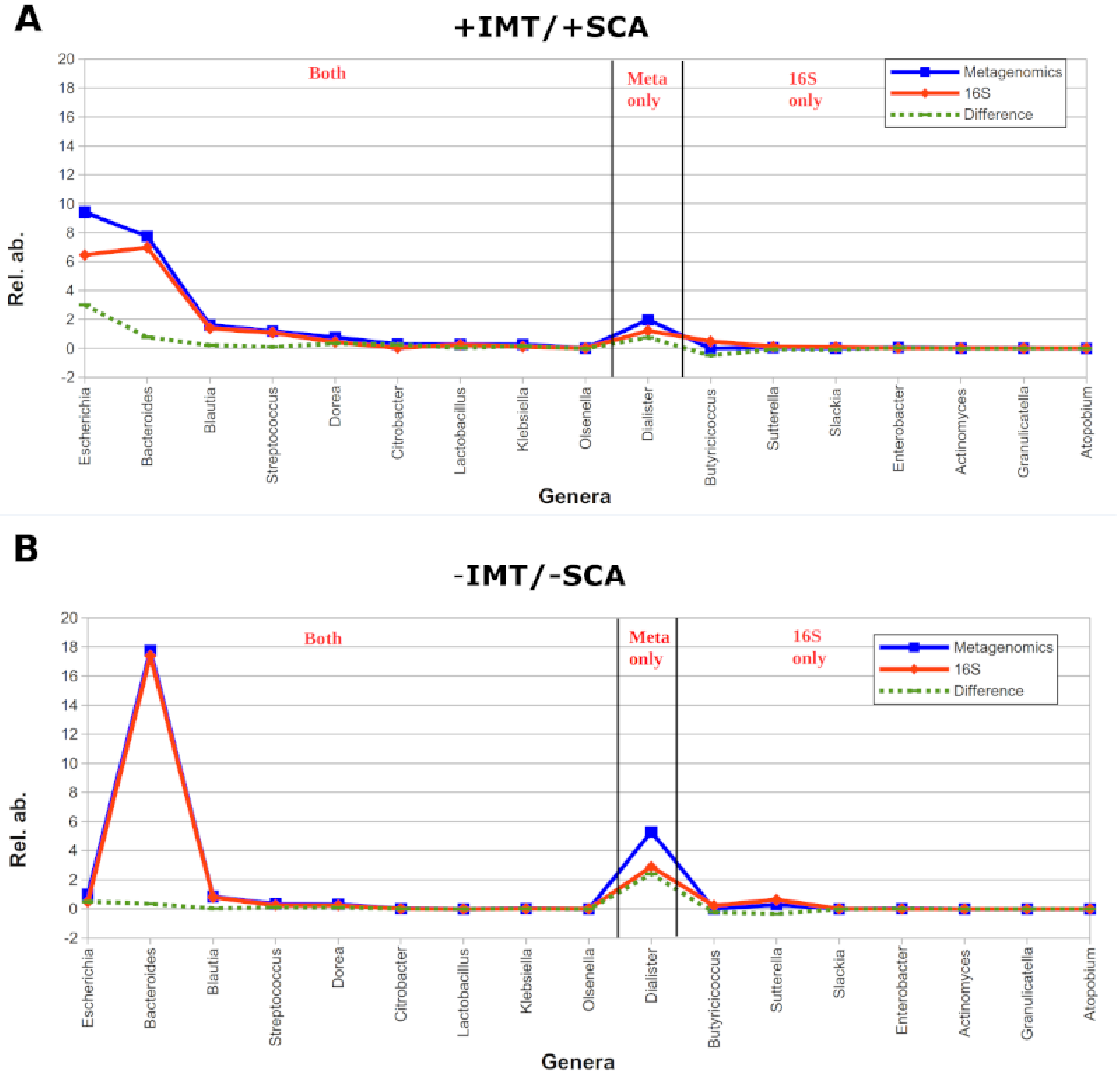

## Supplemental Figure 3 - Taxonomic markers associated with +IMT/+SCA.

(A) PCoA plot of the weighted Unifrac distances among samples; samples were divided in four categories: +IMT/+SCA and -IMT/-SCA correspond to samples that underwent shotgun metagenome sequencing; in order to highlight differences in the PCoA between these two groups, data points are represented with bigger marks and SEM ellipses are shaded. (B) Boxplot of the relative abundances of four genera correlated to IMT and resulting statistically different between –IMT/-SCA (n=23) and +IMT/+SCA (n=23) categories. Categories were determined according to the criteria for the metagenomic sequencing sample selection. Median values are reported as white lines, whereas means are in black. Stars indicates a significant difference (p<0.05, Mann-Whitney test).

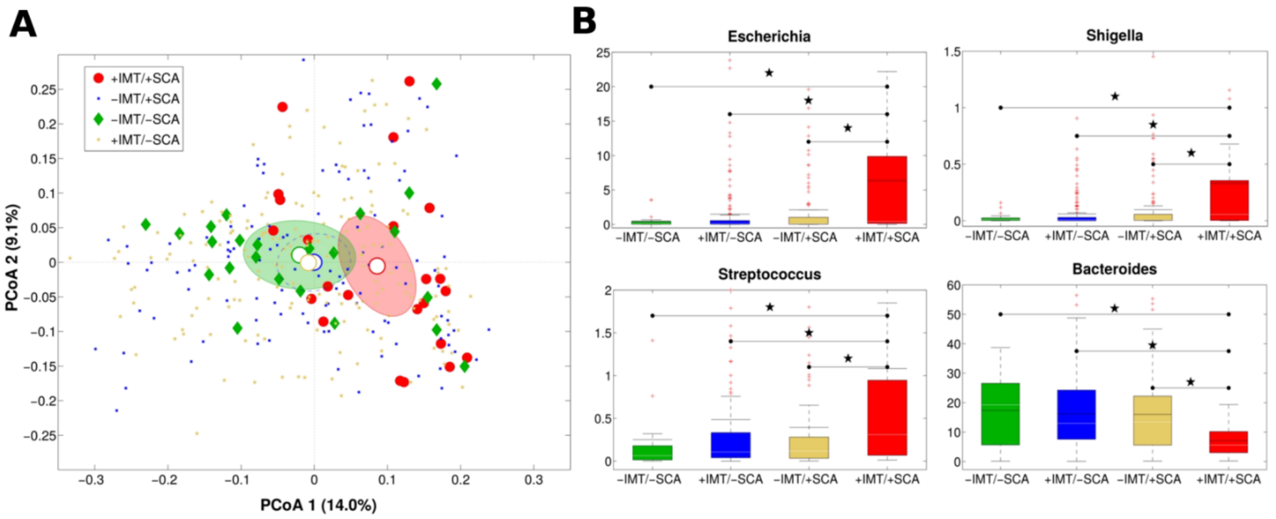

**Supplemental Figure 4 – Correlation between most abundant bacteria and cardio-metabolic markers according to SCA.**

Heatmap of the Spearman's correlation coefficients between bacterial genera relative abundances and cardiovascular/cardio-metabolic risk factors. Correlations were calculated for individuals belonging to "SCA" (n=144) and "no SCA" (n=201) separately. Only genera showing at least one significant correlation (p-value of the linear model <0.05) were represented.

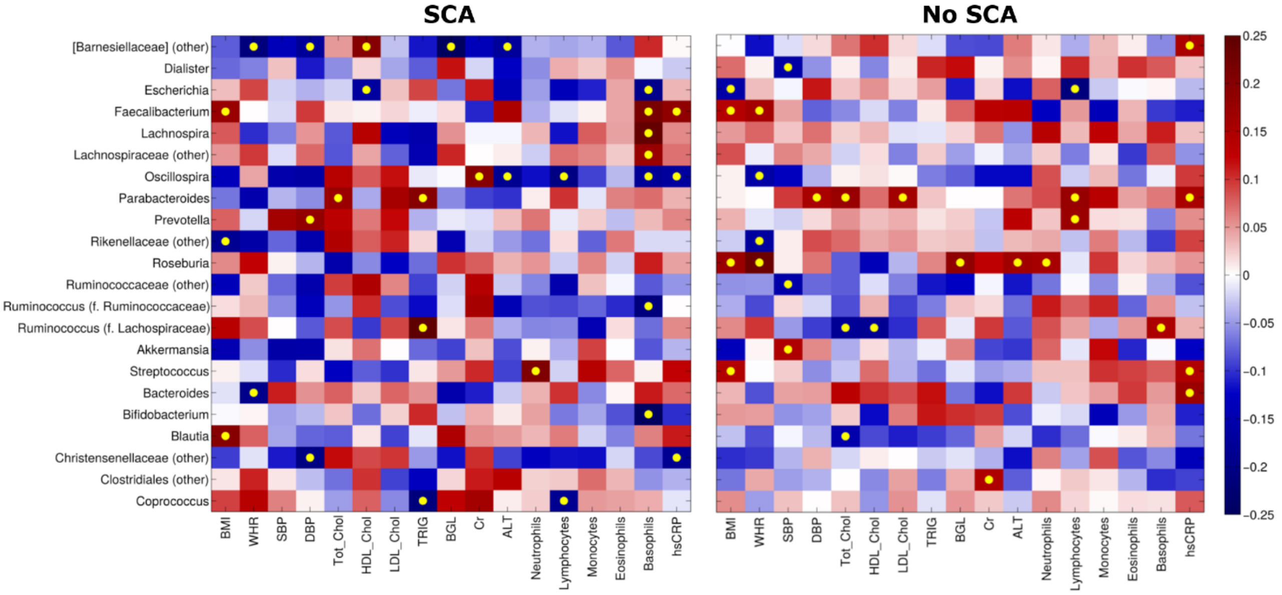

**Supplemental Figure 5 – E. coli metagenetic markers associated with advanced SCA progression.**

(**A**) Genomic alignments of two metagenomic-derived samples on the reference E. coli MG1655genome
(ftp://ftp.ncbi.nlm.nih.gov/genomes/all/GCF/000/005/845/GCF_000005845.2_ASM584v2). Samples were chosen as representative of "-IMT/-SCA" (top) and "+IMT/+SCA" (bottom) categories in order to show the different depth of coverage caiTABCDE operon genes. Metagenomic reads were aligned on reference genome by bwa with default parameters and selecting only reads with ≤ 1 mismatch and ≥ 70 nt matching. Lowest panel reports the genomic annotation.

(**B**) Relative abundances of caiD and caiE genes in the –IMT/-SCA group respect to the +IMT/+SCA group in shown by the boxplot distribution of values; the asterisk marks the significant difference ($p<0.05$).

(**C**) Barplot showing percentages of subjects in the +IMT/+SCA group according to criteria of advanced SCA (see **Supplemental Material** for technical details). White portion represent percentage of subjects with SCA causing stenosis between 30 and 50%; grey portion repent percentage of subjects with SCA causing between 50 and 70% of stenosis of the vascular lumen; black portion represent the percentage of subjects (n=1 out of 23) with SCA causing >70% stenosis of the vascular lumen. P-value is derived from chi square test.

(**D**) Bars and dots plots showing *E.Coli* DNA abundances, using caiE and caiD genes as markers, in fecal samples from subjects in the +IMT/+SCA group divided by advanced (red dots) and no advanced SCA (blue dots) (see **panel C** and **Supplemental Material** for technical details). "*": indicates significant difference, referring to P values from the two-sided Mann-Whitney U-test.

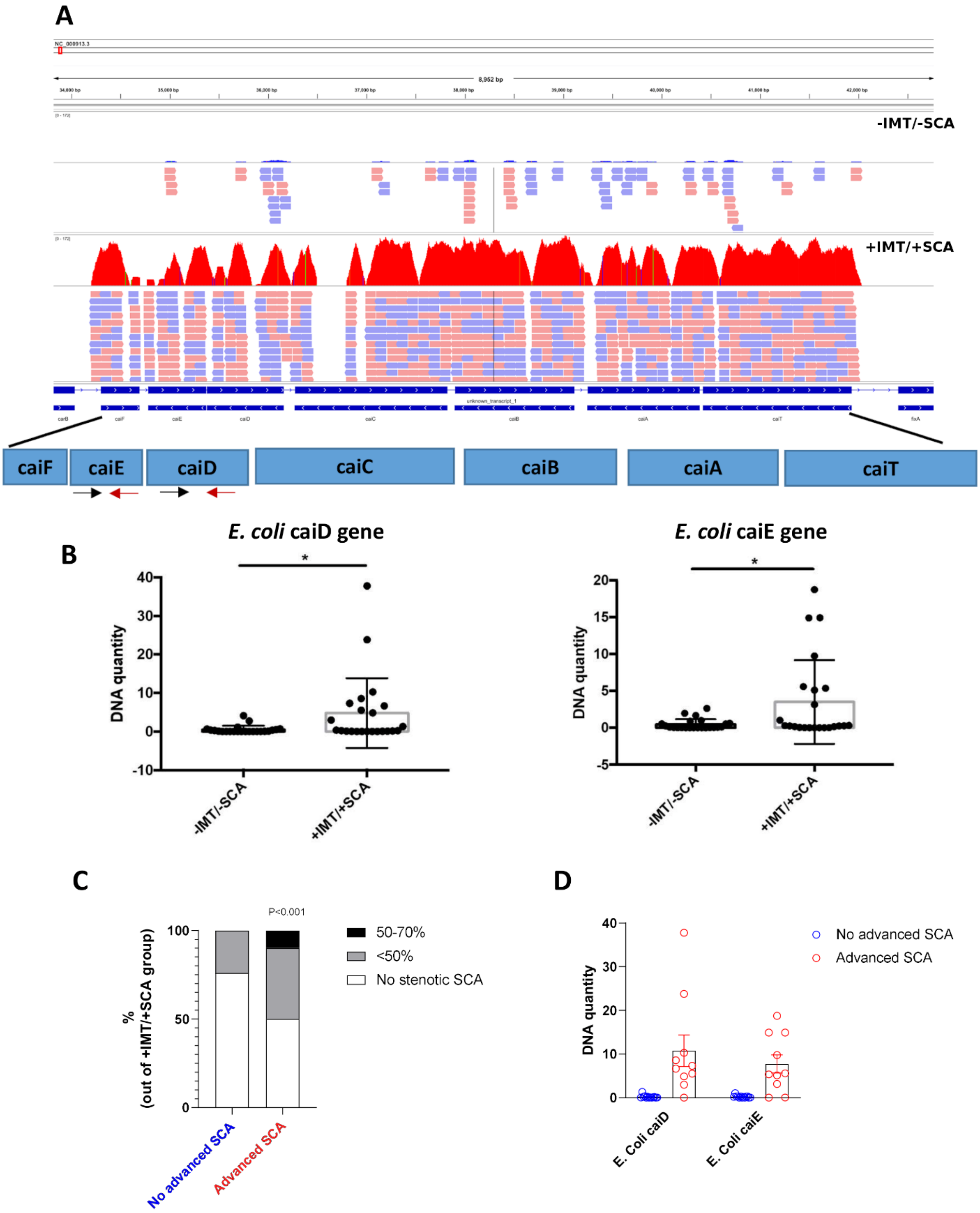
A
-IMT/-SCA
+IMT/+SCA
caiF
caiE
caiD
caiC
caiB
caiA
caiT
B
E. coli caiD gene
E. coli caiE gene
DNA quantity
-IMT/-SCA
+IMT/+SCA
*
C
P<0.001
% (out of +IMT/+SCA group)
50-70%
<50%
No stenotic SCA
No advanced SCA
Advanced SCA
D
DNA quantity
No advanced SCA
Advanced SCA
E. Coli caiD
E. Coli caiE

**Supplemental Figure 6 – Zonulin plasma levels are not increased in subjects with +IMT/+SCA.**

Bars and dot plots showing zonulin levels (ng/mL) from plasma of subjects with +IMT/ +SCA vs subjects with –IMT/-SCA. Bars report mean with standard error.

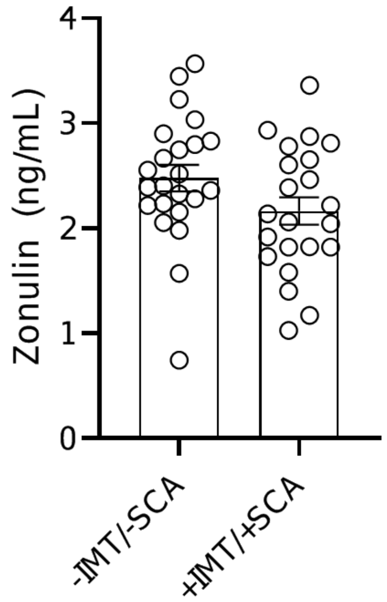

Supplement: Supplementary file 1 [file nutrients-13-00304-s001.pdf]
